# Recent advances to combat ESKAPE pathogens with special reference to essential oils

**DOI:** 10.3389/fmicb.2022.1029098

**Published:** 2022-12-06

**Authors:** Sujogya Kumar Panda, Silvia Buroni, Shasank Sekhar Swain, Andrea Bonacorsi, Erika Alves da Fonseca Amorim, Mukta Kulshrestha, Luis Cláudio Nascimento da Silva, Vishvanath Tiwari

**Affiliations:** ^1^Centre of Environment Studies, Climate Change and Public Health, RUSA 2.0, Utkal University, Vani Vihar, Bhubaneswar, Odisha, India; ^2^Department of Biology and Biotechnology, University of Pavia, Pavia, Italy; ^3^Division of Microbiology and Noncommunicable Diseases (NCDs), Indian Council of Medical Research (ICMR)–Regional Medical Research Centre, Bhubaneswar, Odisha, India; ^4^Laboratory of Microbial Pathogenesis, Universidade Ceuma, São Luís, Brazil; ^5^Department of Biochemistry, Central University of Rajasthan, Ajmer, Rajasthan, India

**Keywords:** antibiotics, biofilm, ESKAPE, essential oils, quorum-sensing, synergy, toxicity

## Abstract

Biofilm-associated bacteria, especially ESKAPE pathogens (*Enterococcus faecium*, *Staphylococcus aureus*, *Klebsiella pneumoniae*, *Acinetobacter baumannii*, *Pseudomonas aeruginosa*, and *Enterobacter* spp.), are a serious challenge worldwide. Due to the lack of discovery of novel antibiotics, in the past two decades, it has become necessary to search for new antibiotics or to study synergy with the existing antibiotics so as to counter life-threatening infections. Nature-derived compounds/based products are more efficient than the chemically synthesized ones with less resistance and lower side effects. In this descriptive review, we discuss the most promising therapeutics for the treatment of ESKAPE-related biofilms. The first aspect includes different types of natural agents [botanical drugs, essential oils (EOs), antimicrobial peptides, bacteriophages, and endolysins] effective against ESKAPE pathogens. The second part of the review deals with special references to EOs/essential oil components (EOCs) (with some exclusive examples), mode of action (via interfering in the quorum-sensing pathways, disruption of biofilm and their inhibitory concentrations, expression of genes that are involved, other virulence factors), existing in literature so far. Moreover, different essential oils and their major constituents were critically discussed using *in vivo* models to target ESKAPE pathogens along with the studies involving existing antibiotics.

## Introduction

**FIGURE 1:**
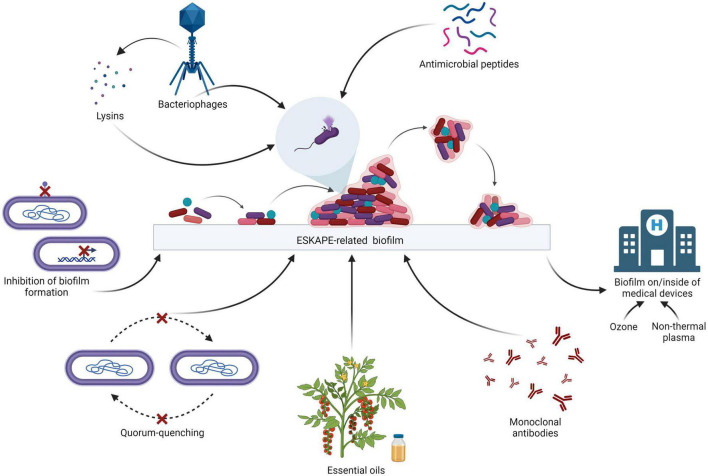
Therapeutic strategies for the treatment of the ESKAPE biofilms (adapted from “Biofilm Formation Cycle”, by BioRender.com, 2022).

The ESKAPE bacteria are a group of opportunistic pathogens consisting of *Enterococcus faecium, Staphylococcus aureus*, *Klebsiella pneumoniae*, *Acinetobacter baumannii*, *Pseudomonas aeruginosa*, and *Enterobacter* species. These bacteria represent a global threat from a clinical point of view since they are generally multidrug-resistant (MDR), extensively drug-resistant (XDR), and pan drug-resistant (PDR). In particular, *E. faecium* is a Gram-positive generally inhabiting the human gastrointestinal tract which may lead to several diseases such as bacteremia, endocarditis, and neonatal meningitis. *S. aureus* is a Gram-positive bacterium colonizing humans at the level of the skin and the upper respiratory tract (nostrils) which maybe involved in skin infections, as well as pneumonia and sepsis. *K. pneumoniae* is a Gram-negative that is part of the normal microbiota of humans (skin and digestive system) which may cause respiratory and urinary infections, as well as bacteremia and liver abscess. *A. baumannii* is a ubiquitous Gram-negative bacterium which may lead to respiratory and urinary infections. *P. aeruginosa* is a ubiquitous Gram-negative causing several infections, including respiratory (especially in cystic fibrosis patients), urinary and skin infections (generally after a burn injury). Last, the genus *Enterobacter* comprises Gram-negative bacteria that may be natural commensals of the human gastrointestinal tract which may be involved in urinary, respiratory and soft skin infections. Due to the resistance of the ESKAPE bacteria to a broad range of antibiotics, there are severe challenges in the treatment of their infections, especially when biofilms are involved. In fact, bacteria inside the biofilms are about 1,000 times more resistant to antimicrobials as compared to planktonic cells (Patil et al., 2021). Consequently, there is an urgent need to develop new weapons to fight these pathogens, with particular emphasis on the eradication of their biofilms (Panda et al., 2021). Several strategies are being explored around the world in order to treat ESKAPE-related biofilms (Sahoo et al., 2021; Figure 1). A broad variety of proteins are involved in biofilm structuration, making them attractive targets to inhibit biofilm formation. For instance, the *Acinetobacter baumannii* outer membrane protein A (OmpA), involved in the virulence of this bacterium, plays a role in the formation of the biofilm: by down-regulating the expression of this protein through synthetic small compounds inhibiting the *ompA* promoter, the *in vitro* formation of biofilm is also affected (Na et al., 2021). Another example is the *Pseudomonas aeruginosa* carbohydrate-binding protein Lectin A (LecA) which is involved in the generation of the biofilm matrix: this molecule may be targeted by LecA synthetic inhibitors in order to impair the structure of the biofilm (Siebs et al., 2022).

For the establishment of biofilm, bacteria have to communicate among each other by means of quorum-sensing. The inhibition of quorum-sensing, i.e., quorum-quenching, is one of the promising strategies to impair biofilm formation. For instance, the *P. aeruginosa* acylase PvdQ is able to cleave the acyl homoserine lactones of *A. baumannii* which are the mediators of quorum-sensing in this pathogen, affecting bacterial communication and, as a consequence, impairing biofilm formation *in vitro* (Vogel et al., 2022).

Other strategies used to target biofilms involve immunotherapy. Besides vaccines (active immunotherapy), monoclonal antibodies (passive immunotherapy) may be exploited to fight the ESKAPE-related biofilms. For example, a monoclonal antibody which targets alginate produced by *P. aeruginosa*, an exopolysaccharide involved in biofilm structuration and protection of the bacterium from the host immune system, could be used. Indeed, when treated *in vitro* with this antibody, *P. aeruginosa* showed impairment in the formation of biofilm (Gao et al., 2020).

In general, it is important to consider both drug reuse/resensitization and drug repurposing. Belonging to the first category, carboxylic acid salts derived from the fluoroquinolone norfloxacin have a higher activity on the ESKAPE pathogens compared to the parent molecule and are active on their biofilms, particularly on the Gram-negative ones (Lowrence et al., 2018). Ethyl bromopyruvate, in spite of being a derivative of anticancer agent 3-bromo-pyruvic acid, can be used for drug repurposing (Kumar et al., 2019). In fact, it is effective on planktonic ESKAPE bacteria and on *Staphylococcus aureus* biofilms, and hence is a good example of drug repurposing (Kumar et al., 2019).

Some of the most promising therapeutics for the treatment of the ESKAPE-related biofilms are antimicrobial peptides, bacteriophages, bacteriophage-encoded products, and natural products such as essential oils (EOs) to eradicate them. It is crucial to consider the treatment of the biofilms of the ESKAPE pathogens on/inside of medical devices since they are sources of nosocomial infections, before implementing the treatment options. For instance, non-thermal plasma (NTP) is a partially ionized gas characterized by both antimicrobial and antibiofilm activity on the ESKAPE pathogens that can be used for the disinfection of medical devices as well as hospital surfaces. NTP is more effective on Gram-negative bacteria compared to Gram-positive bacteria (Scholtz et al., 2021). Besides NTP, ozone can be used in order to eradicate ESKAPE-related biofilms from medical tools (Ibáñez-Cervantes et al., 2022).

This descriptive review includes the discussion of the most promising therapeutics for the treatment of ESKAPE-related biofilms. The first aspect covers different types of natural products effective against ESKAPE pathogens. The second part of the review deals with special references to EOs/essential oil components (EOCs) with some exclusive examples, mode of action, and synergy studies. Moreover, different EOs and their major constituents, as well as *in vivo* models to target ESKAPE pathogens were critically discussed.

## Natural molecules against ESKAPE pathogens

Nature has provided a vast source of therapeutic agents along with a wide range of modern drugs which are in current use. These drugs are obtained from traditional medicinal plants as the diversity of biologically active molecules in these plants make them a potent source of medicines (Kothari et al., 2010; Tiwari et al., 2015). It has been reported that approximately 80% of the world’s population, specifically in developing countries, depend on medicinal plants to fulfill their primary health care needs (Akinsulire et al., 2007). The plant secondary metabolites like alkaloids, flavonoids, terpenes, saponins, tannins, etc., possess different medicinal properties (Mors et al., 2000; Tiwari, 2016).

The prolonged hospital stays have resulted in increased risk of medical expenses and mortality due to hospital-acquired infections which majorly occur in immunocompromised patients because of the exposure to ESKAPE pathogens. Therefore, in order to combat the rapid evolution of disease-causing pathogens, finding new antimicrobials is essential. Numerous secondary metabolites, or phytochemicals, that have the ability to prevent disease are known to be produced by plants. The main benefits of plant-derived products make them viable options for medical treatments because of their potential efficacy and minimal to no negative side effects (Pal and Shukla, 2003). Therefore, the discussion of use of such chemicals and extracts produced from plants to combat ESKAPE pathogens is quite significant. Many efficacious drugs can be produced for disease eradication by using these bioactive compounds. About 80% of the total population which majorly includes developing nations is dependent on natural products. Synthetic drugs are gaining popularity these days due to their time effectiveness, refined quality, cost-effectiveness, and quick effect (Ekor, 2014). However, many natural products–derived compounds are in various stages of drug development and have already highlighted the importance as well as the versatility of natural products as a future of novel drug development. Plants are a vital resource for novel drug development and other pharmacologically active compounds it has been observed that there are many drugs that are developed directly or indirectly from plants. According to World Health Organization (WHO), 11% of 252 drugs are considered basic and essential during the start of the 21st century and these drugs exclusively originated from the flowering plant (Veeresham, 2012). Some recent studies against biofilms provide the best example of natural products being effective and safe like synthetic halogenated furanone molecule, a secondary metabolite derivative, is generated from natural furanone produced by the Australian macroalga *Dilsea pulchra*. This substance can hinder bacterial signaling mechanisms and swarm cell movement. Additionally, it was proposed that *D. pulchra* furanones and AHL molecules’ structural similarity affects how putative regulatory proteins interact with AHL molecules by binding competitively to the receptor. In ecologically relevant concentrations, furanone prevents the surface aggregation characteristics of relevant ecological microorganisms. Other common examples of plant-based drugs are Tannic acid, Endolysins (PlyC), and Epigallocatechin gallate (EGCG) which result in the cleavage of peptidoglycan (Payne et al., 2013).

Proteins including enzymes and transporters are the main target of the herbal compounds. Additionally, these active substances could attach to or block the location where the pathogenic elements bind. These herbal medicines can also impact the behavior of biomolecules, or even their expression in a disease-causing condition. By utilizing cutting-edge techniques, their target and method of action can also be determined, hence, lowering the chance of protein conformations that could cause diseases in the future. Plants can be metabolically engineered to produce more antimicrobial chemicals, which may pave the way for the discovery of new therapeutics. Active substances found in plants which change the expression or shape of proteins that cause illness are proved to be effective against drug-resistant microbes (Table 1). Consequently, they can be useful for creating a brand-new medicine to treat illnesses. To develop phytoconstituents as medicines targeting protein conformation, a thorough biochemical and biophysical investigation is required. Before being employed as medicines, phytoconstituents must be examined for their absorption, distribution, metabolism, excretion, and toxicity (ADMET) characteristics, pharmacophore mapping, effectiveness, and safety. However, for screening the antimicrobial compounds extracted from various plants against a wide range of Gram-positive as well as Gram-negative bacteria, the most commonly adopted methods are minimum inhibitory concentration (MIC), disk diffusion assay, and colony forming unit (CFU). According to the literature survey, it was found that few compounds were highly active against Gram-negative bacteria while some other compounds showed high activity against the Gram-positive bacteria.

**TABLE 1:** Plant active compounds and their significant efficacy against various resistant pathogens.

Active compound	Plant (common name)	Botanical name	Active against	MIC (μg/ml)	References
Hexahydroxy diphenoyl ester vescalagin	Purple loosestrife	*Lythrum salicaria* L.	*Acinetobacter baumannii, Pseudomonas aeruginosa, Staphylococcus aureus*	NA NA 62	Becker et al., 2005; Guclu et al., 2014
Geraniol and terpinol-4-ol	Sugandhakokila	*Cinnamomum glaucescens*	MERSA	369	Panda et al., 2020
Diosgenin, smilagenin, β-sitosterol and hydroxy-tyrosol	Kumarika	*Smilax zeylanica*	MERSA	220	Panda et al., 2020
Propylene glycol	N/A	*Syzygium praecox*	MERSA	1,019	Panda et al., 2020
Tetradecanal and hexadecanoic aicid	Charcoal tree	*Trema orientalis*	MDR-S	369	Panda et al., 2020
β-Amyrine, aerosolic acid, Betulinic acid	Java cedar	*Bischofia javanica*	MDR-S	234	Panda et al., 2020
Myricitrin, mearnsatin-3-O-β-D gluco-pyrnoside	Ceylon olive	*Elaeocarpus serratus*	MDR-S	2,768	Panda et al., 2020
Methyl tridecanoate, arborinone, conferamide	Climbing acacia	*Acacia pennata*	MERSA	369	Panda et al., 2020
Terpenoids and steroids	Long-leaf varnish tree	*Holigarna caustica*	MERSA	369	Panda et al., 2020
α-pinene, methyl salicylate, β- cyclocitrol	Orange Jessamine	*Murraya Pennicula*	MERSA	406	Panda et al., 2020
Glycosides and saponins	Buddha coconut	*Plarygota alata*	MDR-S	550	Panda et al., 2020
No phytochemical reported till now	Lavender scallops	*Kalanchoe fedtschenkoi*	*Staphylococcus aureus, Acinetobacter baumannii, Pseudomonas areugenosa*	256	Richwagen et al., 2019
Saponins, polyphenols, tannins, anthrocynins, triterpines	Coastal golden leaf	*Bridelia mircrantha*	*Staphylococcus aureus, Klebsiella pneumoniae, Pseudomonas arugenosa*, MERSA,	128	Ngane, 2019
glucopyranoside	Bichoo	*Martynia annua*	*Klebsiella pneumoniae, Acinetobacter baumannii, Enterrococcus faecalis, Staphylococcus aureus*	256	Khan et al., 2018
Methanol	Stiff Bottlebrush	*Collistemon rigidus*	MERSA	N/A	Subramani et al., 2017
Aloe—emodine, coniine and lupeol	Bitter Aloe	*Aloe ferox*	*Staphylococcus aureus, Pseudomonas aeruginosa*, *Klebsiella pneumoniae*	310	Ghuman et al., 2016
Ellagic acid	Japanese rose	*Rosa rugosa* Thunb.	*A. baumannii*	250	Miyasaki et al., 2013
Terchebulin Chebulagic acid Chebulinic acid Corilagin	Black- or chebulic myrobalan	*Terminalia chebula* Retz.	*A. baumannii*	500 1,000 62.5 1,000	Miyasaki et al., 2013
Norwogonin Baicalin Baicalein	Chinese skullcap	*Scutellaria baicalensis* Georgi	*A. baumannii*	128 NA NA	Chan et al., 2007; Miyasaki et al., 2013
Eugenol	Clove	*Syzygium aromaticum* (L.) Merr. & L.M.Perry	*A. baumannii*	1,250	Johny et al., 2010; Pelletier, 2012
Trans-cinnamaldehyde	Cinnamon (Dalchini)	*Cinnamomum verum* J.Presl	*A. baumannii*	310	Johny et al., 2010; Pelletier, 2012
Carvacro	Oregano	*Origanum vulgare* L.	Biofilms of *S. aureus*, *A. baumannii*	0.015–0.031% v/v 310	Nostro et al., 2007; Johny et al., 2010; Pelletier, 2012
Thymol	Thyme	*Thymus adamovicii* Velen.	*A. baumannii, Staphylococcus epidermidis* biofilms	NA 0.031%, v/v 0.031–0.062%, V/V	Nostro et al., 2007; Johny et al., 2010; Pelletier, 2012
Curcumin	Turmeric	*Curcuma longa* L.	*S. aureus*, *A. baumannii*	125–250 4 (while EGCG is present)	De et al., 2009; Mun et al., 2013; Betts and Wareham, 2014
Epigallocatechin gallate (EGCG)	Green tea	*Camellia sinensis* (L.) Kuntze	*S. aureus*, *A. baumannii*	100 312–625	Kono et al., 1997; Zhao et al., 2002; Osterburg et al., 2009; Gordon and Wareham, 2010; Betts and Wareham, 2014
Epicatechin	Green tea	*C. sinensis*	*A. baumannii*	NA	Betts et al., 2011
Theaflavin	Black tea	*C. sinensis*	*A.baumannii*	NA NA	Betts et al., 2011
(+)-Lyoniresinol-3 alpha-O-beta-D-glucopyranoside	Chinese boxthorn	*Lycium chinense* Mill.	*A. baumannii*, *S. aureus*, *Enterococcus faecalis*	NA NA NA	Chan et al., 2011
Paeonol	Moutan Peony	*Paeonia* × *suffruticosa* Andrews	*A. baumannii*, *S. aureus*, *E. faecalis*	NA NA NA	Chan et al., 2011
Berberine	Coptidis Rhizoma	*Coptis chinensis* Franch.	*A. baumannii*, *S. aureus*, *E. faecalis*	30	Chan et al., 2011
Berberine	Desert barberry	*Berberis fremontii	*S. aureus*	NA	Stermitz et al., 2000
Honokiol, Magnolol	Cloudforest mangolia	*Magnolia macrophylla* var. *dealbata* (Zucc.) D.L.Johnson	*P. aeruginosa*, *A. baumannii*	NA	Jacobo-Salcedo et al., 2011
α-Elemene, δ-elemene, furanosesquiterpenes	Myrrh	*Commiphora myrrha* (Nees) Engl.	*Klebsiella pneumoniae*, *P. aeruginosa*, *A. baumannii*	6,250 6,250 2,500	Masoud and Gouda, 2012
p-Coumaric acid, ascorbic acid, pyrocatechol, cinnamic acid	Aloe vera	*Aloe barbadensis* Mill.	*S. aureus*, *Streptococcus* sp., *P. aeruginosa*, *K. pneumoniae*	NA	Lawrence et al., 2009
Allyl methyl disulfide, diallylsulfide, diallyltrisulfide, allyl methyl trisulfide, diallyldisulfide, diallyltetrasulfide	Garlic	*Allium sativum* L.	*P. aeruginosa*, *K. pneumoniae*, *A. baumannii*	3,120 1,250 3,120	Khadri et al., 2010
Stigmasterol, nimbiol, sugiol, 4-cymene, α-terpinene, terpinen-4-ol	Neem	*Azadirachta indica* A.Juss.	*S. aureus*, *Enterococci*, *Klebsiella* sp., *P. aeruginosa*	1,000 500 2,000 1,000	Fabry et al., 1998; Nand et al., 2012
Gossypetin, hibiscetin, quercetin, sabdaretin, delphinidin 3-O-sambubioside and cyanidin 3-O-sambubioside	(Indian sorrel/Rose mallow)	*Hibiscus subdarifa Rottb.	*K. pneumoniae*, *Enterobacter aerogens*, *Enterobacter cloacae*	1,024 1,024 256	Djeussi et al., 2013; Rebelo, 2014
Quercetin-7-0-B-Dxylopyranoside, 7-baueren-3-acetate	Baobab/Gorakh imli	*Adansonia digitata* L.	*K. pneumoniae* *E. aerogens* *E. cloacae*	1,024 1,024 1,024	Djeussi et al., 2013

*Unresolved name, NA, MIC information is not available.

### Antimicrobial peptides

One of the most promising alternatives for the treatment of the ESKAPE-related biofilms are the antimicrobial peptides (AMPs). AMPs are positively-charged peptides generally made up of 10–15 amino acids. They are found in all the living organisms and are involved in the innate immunity (Patil et al., 2021). Generally, AMPs are broad-spectrum and cause the osmotic lysis of bacterial cells by permeabilizing their membranes, due to their amphipathic nature. Since this mechanism of action differs from that of antibiotics, resistance to AMPs can be more difficult to achieve (Panda et al., 2021).

Despite their potential, they rarely reach the market mostly due to problems such as low solubility, cytotoxicity, loss of activity (after administration), and susceptibility to proteolysis (Rodríguez et al., 2021; Sarkar et al., 2021). Synthetic molecular evolution can be exploited to evolve the AMPs that may overcome these limitations (Chen et al., 2022). The pipeline starts from parent peptides characterized by a strong antimicrobial activity on both Gram-positive and Gram-negative bacteria *in vitro* and, at the same time, ineffectiveness *in vivo* due to the impediments previously cited. These peptides are modified in order to construct a library of evolved peptides. Then, *In vitro* assays are performed to down-select candidate molecules based on solubility, cytotoxicity and peptide inactivation, besides their antimicrobial activity. Rational variation follows the down-selection step. As an example of an effective evolved AMP, D-CONGA showed a good activity on the ESKAPE pathogens in the planktonic form. Moreover, this peptide dramatically reduced *P. aeruginosa* viability within the biofilm *in vitro* (Starr et al., 2020).

In the context of synthetic AMPs, antimicrobial peptoids are synthetic oligomers that mimic AMPs and are resistant to proteolysis since their backbone is based on nitrogen atoms rather than carbon atoms. Not only their chemical structure is crucial to define the antimicrobial activity, but also their propensity to self-assemble in a physiological environment: among the most interesting therapeutic peptoids, TM peptoids that form bundled or ellipsoidal structures show better antimicrobial and antibiofilm activity against the ESKAPE pathogens compared to the TM peptoids which are not able to properly self-assemble or are characterized by worm-like assemblies. As an example of biologically active TM peptoids, the TM1 peptoid inhibits the growth of planktonic ESKAPE pathogens and affects the formation of their biofilms *in vitro* (Nielsen et al., 2022).

### Bacteriophages and bacteriophage endolysins

Besides AMPs, bacteriophages (phages) and bacteriophage-encoded products can be employed as therapeutics for the treatment of the ESKAPE-related biofilms. Virulent phages are viruses that naturally infect bacterial hosts, eventually causing their lysis. As this interaction is specific, dysbiosis may be prevented. Importantly, they can be used to target antibiotic-resistant strains and are able to eradicate the biofilms of the ESKAPE pathogens. Noteworthy, phages are able to degrade the biofilm matrix (including capsular polysaccharides) via specific enzymes that may promote the access of antibiotics to the deeper regions of the biofilms. Since bacteria can develop resistances against phages, phage cocktails may be used in order to overcome this problem, which also broadens the host range (targeting different strains of the target bacterium). In this context, PA4 is a recently described phage belonging to the *Myoviridae* family which reduces the biomass of *P. aeruginosa* biofilms, impairing the viability of the bacterium *in vitro* (Camens et al., 2021). Similarly, some of the recently characterized vB_SauM kayviruses are able to affect the vitality of *S. aureus* within the biofilm, reducing the pathogen biomass *in vitro* (Kaźmierczak et al., 2022).

During the lytic cycle of the bacteriophage within the bacterial host, lysins play a fundamental role, since they digest the peptidoglycan layer of the bacterial cell wall, causing the lysis of the cell and the release of the phage progeny. One of the features of lysins is that they are generally narrow-spectrum, which means that the beneficial bacteria belonging to the patient’s microbiota are not compromised once administered. Not only lysins are active on planktonic bacteria, but also on biofilms. Efforts have been made in recent years specially to target Gram-negative bacteria. Due to the low permeability of the outer membrane of these bacteria, lysins are not able to reach their target. In this context, LysECD7 is a promising recombinant lysin that shows a promising activity on the Gram-negative ESKAPE pathogens, both in the planktonic form and in the biofilm state, even if a significantly higher lysin concentration is needed for a marked effect on the latter; this lysin is able to cross the outer membrane without any delivery system or additives (Vasina et al., 2021).

Interestingly, lysins encoded by phages infecting Gram-negative bacteria may be characterized by an amphipathic region able to permeabilize the outer membrane after administration, allowing the peptidoglycan layer to be affected. These amphipathic components may be isolated and employed as cationic peptides. PaP1 is a peptide derived from the PlyPa01 lysin (isolated from a phage infecting *P. aeruginosa*) characterized by membrane-acting properties and modified in order to increase the net charge of the peptide, as well as its antibacterial properties. When tested, PaP1 was found active on the ESKAPE pathogens in both monospecies and polymicrobial populations (*A. baumannii*, *P. aeruginosa* and *S. aureus*) and eradicates *P. aeruginosa* mature biofilms *in vitro* (Heselpoth et al., 2022).

### Essential oils

Natural products can be a source of compounds which are active on both the planktonic and sessile forms of bacteria (Da Silva et al., 2017). Plant-derived EOs are a reservoir of volatile, hydrophobic secondary metabolites which may show a broad antimicrobial and antibiofilm activity against microbial pathogens, including the ESKAPE bacteria (El-Tarabily et al., 2021). The antimicrobial properties of these molecules are due to the fact that medicinal and aromatic plants naturally synthetize them in order to respond to various stresses, including microbial attacks. Noteworthy, EOs have a low potential for the development of microbial resistance due to their complex nature with multiple bioactive compounds, that leads to a multi-target activity which is not in the case of conventional antibiotics (Angane et al., 2022).

The mechanisms of action of these compounds are diverse and may be related to the target of either the bacterial virulence factors or the drug resistance mechanisms that characterize these pathogens (Vasconcelos et al., 2018). Belonging to the first category, these metabolites can inhibit biofilm formation and quorum-sensing while, in the second case, they can inhibit the function of efflux pumps and plasmid-mediated resistance (possibly by causing the loss of the plasmid carrying the resistance gene or by interfering with the transfer of the R-plasmid itself to a recipient cell). In the latter cases, EOs can be used in combination with known antibiotics since they mediate re-sensitization of the bacteria to the drug: in this way, the problem of antibiotic resistance can be addressed. It is important to highlight that these metabolites can also have bactericidal properties since they are lipophilic and can alter the membrane permeability, possibly causing membrane disruption (Álvarez-Martínez et al., 2021). An interesting application of EOs is related to their use in the disinfection of medical devices as well as hospital surfaces, preventing hospital-associated infections. As an example of EOs applications in the treatment of the ESKAPE biofilm, *P. aeruginosa* biofilm formation can be impaired *in vitro* by using EOs extracted from Mediterranean plants such as *Foeniculum vulgare* and *Ridolfia segetum* (Artini et al., 2018).

## Anti-biofilm activities of essential oils and/or components

**FIGURE 2:**
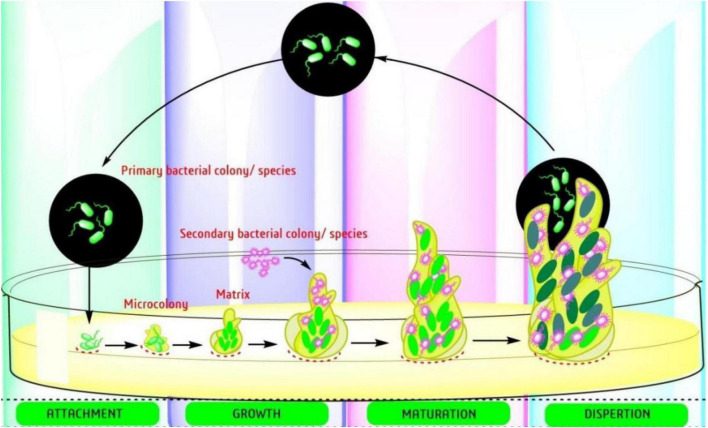
Steps involved in the development of bacterial biofilms (Sahoo et al., 2021).

Biofilm-producing bacteria are typically resistant to antimicrobials and there is an urgent need for new approaches to fill the gap (Jouneghani et al., 2020; Sahoo et al., 2021). The formation of biofilm involves a complex mechanism with several targeted factors. There are several reports already published on the steps involved in development of biofilm (adhesion, microcolonies formation, and maturation) (Figure 2) and the targets starting with the adhesion to mature biofilm. The easiest way to prevent the formation is initiated by attachment, which involves the interaction between adhesive substances and receptors on the host surface. Table 2, provides a list of EOs and their components with anti-biofilm activity against ESKAPE pathogens. The list involves complex oils like Cassia, cinnamon, clove, eucalyptol, lavender, lemon, marjoram, orange, oregano, peppermint, Peru balsam, rosemary, tea, and thyme oil. Moreover, several individual components were also reported including carvacrol, cinnamaldehyde, citral, citronellol, eugenol, Linalool, linalyl acetate, menthol, pulegone, thymol, α-terpineol, and terpinen-4-ol (Table 2). Most detailed studies involved the Gram-positive bacteria *S. aureus*, with genes [*icaD/icaA*, (intracellular gene)], biofilm-associated protein (*bap*), and several controlling genetic loci, e.g., *sarA luxS*, and *agr* quorum sensing (QS) (Yadav et al., 2015). Beside *S. aureus*, the Gram-negative bacteria *P. aeruginosa* is well studied with its mode of action.

**TABLE 2:** Antibacterial activities of essential oils/essential oil components against ESKAPE pathogen.

Essential oils and constituent(s)	Pathogenic bacteria	MIC values; (references)	MBIC; (references)
*Cinnamomum cassia* (L.) J.Presl.	*P. aeruginosa*	–; (Condò et al., 2020)	0.2%, v/v; (Kavanaugh and Ribbeck, 2012)
*Coriandrum sativum* L.	*A. baumannii*	1–4 μl/ml; (Duarte et al., 2013)	4 μl/ml; (Duarte et al., 2013)
*Cymbopogon flexuosus* (Nees ex Steud.) W.Watson	*S. aureus*, MRSA	0.5–4 mg/ml; (Piasecki et al., 2021)	0.06% v/v; (Adukwu et al., 2012)
*Cymbopogon nardus* (L.) Rendle	*S. aureus*	–	0.5 mg/ml; (Pontes et al., 2019)
*Mentha piperita* L.	28 clinic strains of *S. aureus*	64–256 μg/ml; (Li et al., 2011)	–
*Origanum vulgare* L.	*S. aureus*	–	10 μl/ml; (Dos Santos Rodrigues et al., 2018)
*Perilla frutescens* L. Britton	*S. aureus*, MRSA	0.4 μl/ml; (Qiu et al., 2011)	–
*Plectranthus amboinicus* (Lour.) Spreng.	*S. aureus*	0.25 mg/ml; (Vasconcelos et al., 2017)	0.5 mg/ml; (Vasconcelos et al., 2017)
*Syzygium aromaticum* (L.) Merr. & L.M.Perry	*S. aureus*	85.4 μl/ml; (Kačániová et al., 2021)	0.106 mg/ml; (Budri et al., 2015)
*S. aromaticum*	*P. aeruginosa*	223.3 μl/ml; (Kačániová et al., 2021)	1.3% v/v; (Kavanaugh and Ribbeck, 2012)
Cassia oil	*S. aureus*	0.3%, v/v; (Kavanaugh and Ribbeck, 2012)	0.6%, v/v; (Kavanaugh and Ribbeck, 2012)
Cassia oil	*P. aeruginosa*	0.2%, v/v; (Kavanaugh and Ribbeck, 2012)	0.4%, v/v; (Kavanaugh and Ribbeck, 2012)
Cinnamon oil	*K. pneumoniae*, *S. aureus*	>1.6 mg/ml 3.2 mg/ml; (Prabuseenivasan et al., 2006)	*S. aureus* (1 mg/ml) (Cui et al., 2016)
Clove oil	*P. aeruginosa*; *K. pneumoniae* and *S. aureus*	>1.6 mg/ml; >6.4 mg/ml; (Prabuseenivasan et al., 2006)	3.2%, sub MIC, biofilm of *P. aeruginosa;* (Husain et al., 2013)
Clove oil	*S. aureus*	1.2%, v/v; (Kavanaugh and Ribbeck, 2012)	1.6%, v/v; (Kavanaugh and Ribbeck, 2012)
Lavendor oil	*S. aureus*	0.8%, v/v; (Budzyńska et al., 2011)	1.6%, v/v; (Budzyńska et al., 2011)
Lavendor oil	*A. baumannii*	10.5–13.0 μl/ml; (Sienkiewicz et al., 2014)	>5%, v/v (*P. aeruginosa*); (Kavanaugh and Ribbeck, 2012)
Lemon oil	*K. pneumoniae;* *P. aeruginosa, S. aureus*	>12.8 mg/ml; (Prabuseenivasan et al., 2006)	*K. pneumoniae* (170 μl/L); (Sahal et al., 2016)
Lemon Balm oil	*S. aureus*	0.1%, v/v; (Budzyńska et al., 2011)	0.4%, v/v; (Budzyńska et al., 2011)
Orange oil	*K. pneumoniae*, *P. aeruginosa; S. aureus*	≥12.8 mg/ml >6.4 mg/ml; (Prabuseenivasan et al., 2006)	–
Oregano oil	*S. aureus*	0.062%, v/v; (Nostro et al., 2007)	0.5%, v/v; (Nostro et al., 2007)
Peru Balsam oil	*S. aureus*	2.5%, v/v; (Kavanaugh and Ribbeck, 2012)	3.5%, v/v; (Kavanaugh and Ribbeck, 2012)
Peru Balsam oil	*P. aeruginosa*	2.5%, v/v; (Kavanaugh and Ribbeck, 2012)	3.5%, v/v; (Kavanaugh and Ribbeck, 2012)
Rosemary oil	*K. pneumoniae, P. aeruginosa*; *S. aureus*	>6.4 mg/ml; >12.8 mg/ml; (Prabuseenivasan et al., 2006)	–
Tea tree oil	*S. aureus*	0.4%, v/v; (Budzyńska et al., 2011)	0.8%, v/v; (Budzyńska et al., 2011)
Thyme oil	*S. aureus*	0.5%, v/v; (Kavanaugh and Ribbeck, 2012)	1.6%, v/v; (Kavanaugh and Ribbeck, 2012)
Allicin	MSSA and MRSA	32–64 μg/ml; (Leng et al., 2011)	80 μg/ml; (Zhang et al., 2022)
Carvacrol	*S. aureus*	0.35–2.80 mg/ml; (Rosato et al., 2010)	0.25 mg/ml; (Vasconcelos et al., 2017)
Carvacrol	*S. aureus*	0.02%, v/v; (Yadav et al., 2015)	0.04%, v/v; (Yadav et al., 2015)
Carvacrol	*S. aureus*	0.015%, v/v; (Nostro et al., 2007)	0.25%, v/v; (Nostro et al., 2007)
Carvacrol	*S. aureus*	0.3 μl/ml; (Souza et al., 2013)	200 μl/L; (Espina et al., 2017)
Carvacrol	Group A *Streptococci*	64–256 μg/ml; (Magi et al., 2015)	*E. faecalis* (0.01%, v/v); (Campana and Baffone, 2018)
Cinnamaldehyde	*P. aeruginosa*	0.1%, v/v; (Kavanaugh and Ribbeck, 2012)	0.2%, v/v; (Kavanaugh and Ribbeck, 2012)
Cinnamaldehyde	*P. aeruginosa*	–	11.8 mM; (Topa et al., 2018)
Cinnamaldehyde	*S. aureus*	–	0.199 mg/ml; (Budri et al., 2015)
Citral	*S. aureus*, MRSA	0.5 mg/ml; (Porfírio et al., 2017)	500 μl/L; (Espina et al., 2017)
Citronellol	*S. aureus*	0.35–1.40 mg/ml; (Rosato et al., 2007)	0.5 mg/ml; (Piasecki et al., 2021)
Curcumin	*P. aeruginosa*	62.5 μg/ml; (Adamczak et al., 2020)	100 μg/ml; (Packiavathy et al., 2014)
Eucalyptol	*S. aureus*	2.80–5.60 mg/ml; (Rosato et al., 2007)	256 μg/ml; (Hendry et al., 2009)
Eugenol	*S. aureus*	0.04%, v/v; (Yadav et al., 2015)	0.08%, v/v; (Yadav et al., 2015)
Eugenol	*S. aureus* (26 strains)	128–512 μg/ml; (Qiu et al., 2010)	0.237 mg/ml; (Budri et al., 2015)
Geraniol	*S. aureus*	0.08–1.40 mg/ml; (Rosato et al., 2007)	0.25 mg/ml; (Pontes et al., 2019)
Geranium	*K. pneumoniae*, *P. aeruginosa*, *S. aureus*	12.8 to >12.8 mg/ml; (Prabuseenivasan et al., 2006)	–
Limonene	MRSA	–	200 μl/L; (Espina et al., 2015)
Linalool	*S. aureus*	0.2–2.5 mg/ml; (Sonboli et al., 2005)	*A. baumannii* (8 μl/ml); (Alves et al., 2016)
Linalool	*S. aureus*	0.19%, v/v; (Budzyńska et al., 2011)	0.78%, v/v; (Budzyńska et al., 2011)
Linalyl acetate	*S. aureus*	0.19%, v/v; (Budzyńska et al., 2011)	0.19%, v/v; (Budzyńska et al., 2011)
Marjoram	*S. aureus*	0.125% v/v; (El-Shenawy et al., 2015)	Mixed biofilm (0.25 and 20 mg/ml); (Kerekes et al., 2019)
Menthol	*S. aureus*, MRSA	>2 mg/ml; (Qiu et al., 2010)	0.64–1.98 mg/ml; (Kifer et al., 2016)
Peppermint	*S. aureus*	0.5% (v/v); (Xiao et al., 2020)	0.5 mg/ml; (Kang et al., 2019)
Pulegone	*S. aureus*	3.75 to >15 mg/ml; (Sonboli et al., 2006)	–
Thymol	*S. aureus*	0.7–1.40 mg/ml; (Rosato et al., 2007)	0.5 μl/ml; (Ceylan and Ugur, 2015)
Thymol	*S. aureus*	0.031%, v/v; (Nostro et al., 2007)	0.25%, v/v; (Nostro et al., 2007)
Triacetin	*S. aureus*	22.40 mg/ml; (Rosato et al., 2007)	–
α- terpineol	*S. aureus*	0.19%, v/v; (Budzyńska et al., 2011)	0.38%, v/v; (Budzyńska et al., 2011)
Terpinen-4-ol	*S. aureus*	0.19%, v/v; (Budzyńska et al., 2011)	0.19%, v/v; (Budzyńska et al., 2011)

–, data not available; MIC, minimum inhibitory concentration; MBIC, minimum biofilm inhibitory concentration; MSSA, methicillin susceptible S. *aureus*; MRSA, methicillin resistant S. *aureus*.

## Essential oil and quorum sensing—The language of bacteria

The two most studied bacteria are *S. aureus* and *P. aeruginosa*. Two most common oils, viz. clove oil (Husain et al., 2013) and peppermint oil (Husain et al., 2015), were tested to see their effects in reducing *P. aeruginosa* QS regulated biofilm formation. Authors observed a strong effect of both oils along with menthol as a constituent in inhibiting biofilm formation (84%), swarming migration (81%), production of virulence factors like LasB elastase activity (80%), protease activity (76%), chitinase activity (78%), and pyocyanin production (85%) (Table 3). A similar observation was also verified with eugenol on two MDR *P. aeruginosa.* Moreover, the authors found additional virulence factors such as rhamnolipid (57%), and pyoverdine (69%), which are responsible for microbial cell adhesion (Rathinam et al., 2017). Another report is also available by Al-Shabib et al. (2017) on the effect of eugenol to reduce the QS-regulated production of similar virulence factors in *P. aeruginosa* PAO1. In *P. aeruginosa*, curcumin (1 μg/ml) inhibits the formation of signaling molecules (Rudrappa and Bais, 2008). Tea oil was also observed to be effective in controlling biofilm formation in *S. aureus*, with interesting findings by transcriptome analysis as 104 genes downregulated while 200 genes were upregulated. Many of these genes are linked to biofilm formation, e.g., *sarA* gene (encodes the DNA-binding protein SarA) which is downregulated, and responsible for biofilm (Zhao et al., 2018).

**TABLE 3:** Mode of action of select EOs/EOC against ESKAPE pathogen.

Essential oils	Pathogen	Mode of action	References
*Chenopodium ambrosioides*/α-Terpinene	*S. aureus* (IS-58)	Inhibition of EPs (*tetK*)	Limaverde et al., 2017
*C. ambrosioides*/α-Terpinene	*S. aureus* (1199B/1199)	Inhibition of EPs (*norA*)	Salehzadeh et al., 2018
*C. verum*	*K. pneumoniae*	Induction of oxidative stress and oxidation/DBM	Yang et al., 2019
*C. cyminum*	*K. pneumoniae*	Loss of plasmid integrity	Derakhshan et al., 2008
*Dodartia orientalis*	*S. aureus*	DBM	Wang et al., 2017
*Dorema aucheri* Bioss	*P. aeruginosa* PAO1	QS-inhibition, reduction on virulence factors (RVF) (pyoverdine and elastase production), and the transcription of *lasI*	Sepahi et al., 2015
*Ferula asafoetida* L.	*P. aeruginosa* PAO1	QS-inhibition, RVF (pyocyanin, pyoverdine, elastase production), and the transcription of lasI	Sepahi et al., 2015
*Melaleuca alternifolia* (tea tree oil)	*P. aeruginosa* NCTC 6749	DBM and metabolic events	Cox et al., 2001
*Melaleuca alternifolia*	Methicillin-resistant *S. aureus*, Carbapenem-resistant *K. pneumoniae, A. baumannii*, and *P. aeruginosa*	DBM (structural integrity and membrane permeabilization)	Oliva et al., 2018
Tea tree oil	*S. aureus*	Cell division inhibition	Reichling et al., 2002
*Mentha piperita* L.	*S. aureus*	Inhibition of α-toxin production (ITP)	Li et al., 2011
*Mentha piperita* L.	*P. aeruginosa* PAO1	QS-RVF (elastase, proteases, pyocyanin and chitinase)	Husain et al., 2015
*Ocimum gratissimum*	*P. aeruginosa* and *S. aureus*	Change in membrane permeability (CMP)	Hyldgaard et al., 2012
*Origanum vulgare* (oregano oil)	*P. aeruginosa* and *S. aureus*	Bacterial enzyme inhibition and reduce bacterial lipase and coagulase activity	Lambert et al., 2001
*Rosmarinus officinalis*/1,8-cineol	MDR *K. pneumoniae*	CMP	Moreno et al., 2015
*Rosmarinus officinalis*/Eucalyptol	MDR *A. baumannii* and *P. aeruginosa*	Inhibition of EPs	Saviuc et al., 2016
*Origanum vulgare* L.	*S. aureus*	ITP	De Souza et al., 2010
*Perilla frutescens* L. Britton	*S. aureus*, MRSA	ITP	Qiu et al., 2011
*Syzygium aromaticum*/Eugenol	MRSA	Anti-QS and anti-biofilm	Al-Shabib et al., 2017
*Syzygium aromaticum*	*P. aeruginosa*	QS- Inhibit swarming motility	Khan et al., 2009
*Thymus vulgaris*/thymol	*S. aureus*	Inhibition of EPs (norA)	Salehzadeh et al., 2018
*Essential oil components*			
Allicin	MSSA and MRSA	ITP	Leng et al., 2011
Carvacrol	*S. aureus*	DBM	Di Pasqua et al., 2007
Carvacrol	*S. aureus*	Inhibit enterotoxin production completely	Souza et al., 2013
Carvacrol	MRSA	Anti-QS and anti-biofilm	Sharifi et al., 2018
Carvacrol	*S. aureus*	Changes in fatty acid composition	Noumi et al., 2018
Carvacrol	*P. aeruginosa*	QS-Inhibition (pyocyanin production, biofilm)	Tapia-Rodriguez et al., 2017
Carvone	MRSA	Disruption and separation of the cytoplasmic contents by R-cat	Oosterhaven et al., 1995
Cinamoldehyde	Carbapenem resistant *A. baumannii*	Anti-biofilm	Mohamed et al., 2018
Cinamoldehyde	*P. aeruginosa* PA01	QS-Inhibition 7-fold of the lasR level, and pyocyanin production	Ahmed et al., 2019
Curcumin	*P. aeruginosa* PA01	QS-Inhibition 2-fold of elastase activity	Rudrappa and Bais, 2008
Curcumin	*P. aeruginosa* PA01	QS-Inhibition (alginate and prodigiosin production)	Packiavathy et al., 2014
Eugenol	*S. aureus*	Inhibit enterotoxin and α-hemolysin production significantly	Qiu et al., 2010
Farnesol and nerolidol	*S. aureus*	Cytoplasm disruption (alteration of integrity and permeability, inhibition of cell respiration, K^+^ leakage)	Cox et al., 2000; Inoue et al., 2004
6-Gingerol	*P. aeruginosa*	QS-Inhibition (biofilm formation, 53%), RVF (exoprotease, pyocyanin and rhamnolipid)	Kim et al., 2015
Linalyl acetate	*S. aureus*	DBM and leakage of intracellular materials	Trombetta et al., 2005; Mahizan et al., 2019
Linalyl anthranilate	Carbapenemase-producing *K. pneumoniae*	DBM and leakage of intracellular materials	Yang et al., 2021
Menthol	*S. aureus*	DBM and leakage of intracellular materials	Trombetta et al., 2005; Mahizan et al., 2019
Menthol	*S. aureus*, MRSA	ITP	Qiu et al., 2010
Menthol	*P. aeruginosa* PA01	QS-Inhibition, Anti-biofilm, RVF (protease activity, elastase activity, chitinase activity, pyocyanin production, swarming motility, EPS production)	Husain et al., 2015
β-pinene	*E. faecalis*	Anti-biofilm	Negreiros et al., 2016
Thymol	*S. aureus*	DBM and leakage of intracellular materials	Trombetta et al., 2005; Mahizan et al., 2019
Thymol	MRSA	Anti-biofilm and anti-quorum sensing	Sharifi et al., 2018
Thymol	*S. aureus*; MRSA	Inhibit α-hemolysin, enterotoxin production	Qiu et al., 2011
α- and γ-terpinene	*S. aureus*	DBM	Mahizan et al., 2019
Terpinene-4-ol	MRSA	Anti-QS and anti-biofilm	Perez et al., 2019
Terpinene-4-ol	*S. aureus*	Formation of multilamellar, mesosome-like structures	Carson et al., 2002; Reichling et al., 2002
Zingerone	*P. aeruginosa* PA01	QS-Inhibit protease production	Kumar et al., 2015

CMP, change in membrane permeability; DBM, disruption of bacterial membrane; Eps, extracellular polymeric substances; ITP, inhibition of α-toxin production; QS, quorum sensing.

Eugenol is also very effective in reducing the formation of biofilm in methicillin-resistant *S. aureus* (MRSA) (MIC: 0.04%) (Yadav et al., 2015). With the help of RT-qPCR tests, it was confirmed that eugenol (0.5 × MIC) reduced *sarA*, *seA* and *icaD* expression. Further, the gene expression studies showed why eugenol is strong enough to reduce the biofilm since these genes are related to biofilm e.g. regulatory gene (*sarA*), enterotoxin gene (*seA*), and adhesion gene (*icaD*) (Yadav et al., 2015). The authors also studied the effects of eugenol in *in vivo* experiments using an otitis media rat model. Eugenol (0.02%) bears a significant reduction of the bacterial colonization without any biofilms in comparison to the control tympanic bulla which was filled with cell debris and biofilm (Yadav et al., 2015). Another study also evidenced a significant reduction of biofilm in MRSA at sub-MIC concentration (Al-Shabib et al., 2017).

Several monoterpenes (citral, MIC = 500 μl/L; carvacrol, MIC = 200 μl/L; (+)-limonene, MIC = 5,000 μl/L), also showed reduction of biofilm formation in *S. aureus* at sub-MIC concentration. Among them, carvacrol is reported to be highly potent with the lowest concentration of 10 μl/L up to 80% reduction (Espina et al., 2015). In *P. aeruginosa*, cinnamaldehyde is able to reduce the production of AHLs probably due to hydrophobic interaction and strong bonding between cinnamaldehyde and LasI (Chang et al., 2014).

## Eradication of established biofilms

Most of the biofilm studies are performed using spectrophotometer for absorption analysis performed using biofilm specific dyes. These assays indicate only the inhibition of biofilms and do not indicate the capability of the compound to eradicate an already established biofilm. All ESKAPE pathogens are biofilm-associated and, due to their intrinsic resistance, they are difficult to treat. Table 2 shows a list of selected essential oils/essential oil components and their activity against ESKAPE pathogens in both planktonic (MIC values) and biofilm cells (Minimum Biofilm Inhibitory Concentration, MBIC). Most studies are found to be effective against *S. aureus* followed by *P. aeruginosa* while limited studies have been performed on *A. baumannii* and *K. pneumoniae.* In most cases, it is observed that the MIC value is not sufficient to inhibit biofilm, while in few cases even 100 fold could is not sufficient to treat established biofilm. Coelho and Pereira (2013) tested the three most common oils, e.g., cinnamon, tea, and palm rosa on established biofilm of *P. aeruginosa*. The authors noticed a sufficient reduction of the bacterial population (5 log10 to 2.5 log10 CFU/cm^2^) and compared it with the standard antibiotic ciprofloxacin (Coelho and Pereira, 2013). Another interesting study by Lu et al. (2018) established the removal of preformed biofilm against both *S. aureus* and *P. aeruginosa* biofilms when treated with the oregano oil (carvacrol as a major constituent) (Lu et al., 2018). The experiment was further validated by an *in vivo* mouse model (third-degree burn wound infection) treated with the oregano oil (10 mg/ml for 3 days), resulting in a reduction of the 3 log10 steps population (Lu et al., 2018). Moreover, authors also detected changes in the cell structure, with disruption of biofilm under electron microscopy. This kind of study is highly valuable as it shows the direct effect of EOs/EOC and can distinguish the destruction or eradication of established biofilm.

## Bactericidal toxic effects of essential oils and essential oil components on biofilms

Only limited oils/components were reported to be bactericidal. Eugenol is one of the major components that act very effectively at higher concentrations, at 12.8% (v/v), with 91.6% on the inhibitions of biofilms formed by MDR *S. aureus*. In comparison to eugenol, thyme oil also showed similar antibiofilm activity. The highest biofilm reduction (88% inhibition of *S. aureus* JSA10) was observed at 12.8% v/v (Jafri et al., 2014). Both *S. aureus* and *P. aeruginosa* were inhibited significantly when treated with cinnamaldehyde, a major constituent of *Cinnamomum zeylanicum* and *Cinnamomum cassia.* An interesting observation made by the authors is that the inhibition is not due to its anti-QS effect, but to its cytotoxic effects (Firmino et al., 2018).

## Synergistic potentials of essential oils/essential oil components along with antibiotics against multidrug-resistant ESKAPE pathogens

In the past decade, there has been an increase in the research on the synergetic potentials of clinical antibiotics in combination with essential oils or individual components. We have reviewed various publications on the ESKAPE pathogens and summarized (Table 4) the synergistic effects of essential oils and antibiotics.

**TABLE 4:** Synergistic effects of Essential oils and antibiotics against ESKAPE pathogens.

EOs/EOC	Antibiotics	Bacterial strains	Method(s)	Outcome	Mechanism of action	References
*Artemisia herba-alba* Asso	Chloramphenicol	*E. aerogenes* EA27	Microdilution method (MDM)	4-fold reduction in MIC	Alteration of outer membrane (OM), lipopolysaccharide structure (LPS)	Fadli et al., 2016
*Aniba rosaeodora*	Gentamicin	*A. baumannii*	Checkerboard assay (CBA)	FICI = 0.11	–	Rosato et al., 2007
*Cymbopogon citratus* (DC.) Stap	Chloramphenicol	*E. aerogenes* EA27	MDM	4-fold reduction in MIC	Alteration of OM-LPS	Fadli et al., 2016
*Cinnamomum zeylanicum*	Amikacin	*Acinetobacter* sp.,	CBA	Additive effect	–	Guerra et al., 2012
*Coriandrum sativum*	Chloramphenicol, Ciprofloxacin, Gentamicin	*A. baumannii*	CBA	FICI = 0.047 to 0.375	–	Duarte et al., 2012
*Croton zehntneri* Pax & K.Hoffm	Norfloxacin	*S. aureus* SA 1199B	Change in inhibition zone by EO gaseous contact (IZGC)	IZGC = 39.5%	Inhibition of efflux pumps activity/expression (IEPA/E)	Coutinho et al., 2010
*Helichrysum italicum* (Roth) G.Don	Chloramphenicol	*E. aerogenes* EA27	MDM	8-fold reduction in MIC	IEPA/E	Lorenzi et al., 2009
*Helichrysum italicum* (Roth) G.Don	Chloramphenicol	*A. baumannii* AP1	MDM	8-fold reduction in MIC	IEPA/E	Lorenzi et al., 2009
*Lippia microphylla* Cham.	Norfloxacin	*S. aureus* SA 1199B	IZGC	IZGC = 39.5%	IEPA/E	Coutinho et al., 2011
*Myrtus communis*	Ciprofloxacine, polymixin B	*A. baumannii*	CBA	FICI = 0.047–0.375	–	Aleksic et al., 2014
*Origanum vulgare* L	Tetracycline	*S. aureus* I-58	MDM	4-fold reduction in MIC	IEPA/E	Cirino et al., 2014
*Pelargonium graveolens*	Gentamicin	*A. baumannii, S. aureus*	CBA	FICI = 0.11	–	Rosato et al., 2007
*P. graveolens*	Norfloxacin	*S. aureus*	CBA	FICI = 0.11	–	Rosato et al., 2007
*Thymus broussonetii* Bois	Cefixime	MDR *P. aeruginosa*	CBA, Cell lysis assay (CLA)	FICI = 0.5	Disruption of bacterial membrane (DBM)	Fadli et al., 2012
*T. broussonetii*	Cefixime	MDR *S. aureus*	CBA; CLA	FICI = 0.5	DBM	Fadli et al., 2012
*T. broussonetii*	Ciprofloxacin	MDR *P. aeruginosa*	CBA, CLA	FICI = 0.14	DBM	Fadli et al., 2012
*T. broussonetii*	Ciprofloxacin	MDR *S. aureus*	CBA, CLA	FICI = 0.5	DBM	Fadli et al., 2012
*T. broussonetii*	Gentamycin	MDR *E. clocae*	CBA, CLA	FICI = 0.5	DBM	Fadli et al., 2012
*T. broussonetii*	Gentamycin	MDR *P. aeruginosa*	CBA, CLA	FICI = 0.28	DBM	Fadli et al., 2012
*T. broussonetii*	Gentamycin	MDR *S. aureus*	CBA, CLA	FICI = 0.5	DBM	Fadli et al., 2012
*T. broussonetii*	Pristinamycin	MDR *K. pneumoniae*	CBA, CLA	FICI = 0.5	DBM	Fadli et al., 2012
*T. broussonetii*	Pristinamycin	MDR *S. aureus*	CBA, CLA	FICI = 0.5	DBM	Fadli et al., 2012
*Thymus maroccanus* Ball	Chloramphenicol	*A. baumannii* AP1	MDM	8 to 16 -fold reduction in MIC	Alteration of OM- LPS	Fadli et al., 2011
*T. maroccanus*	Chloramphenicol	*P. aeruginosa* PA124	MDM	4 to 8 -fold reduction in MIC	IEPA/E	Fadli et al., 2011
*Thymus riatarum* Humbert & Maire	Chloramphenicol	*E. aerogenes* EA27	MDM	16 -fold reduction in MIC	IEPA/E	Fadli et al., 2011
*T.riatarum*	Ciprofloxacin	MDR *E. clocae*	CBA, CLA	FICI = 0.37	DBM	Fadli et al., 2012
*T. riatarum*	Ciprofloxacin	MDR *K. penumoniae*	CBA, CLA	FICI = 0.5	DBM	Fadli et al., 2012
*T. riatarum*	Ciprofloxacin	MDR *P, aeruginosa*	CBA, CLA	FICI = 0.15	DBM	Fadli et al., 2012
*T. riatarum*	Ciprofloxacin	MDR *S. aureus*	CBA, CLA	FICI = 0.26	DBM	Fadli et al., 2012
*T. riatarum*	Gentamycin	MDR *E. clocae*	CBA, CLA	FICI = 0.19	DBM	Fadli et al., 2012
*T. riatarum*	Gentamycin	MDR *K. penumoniae*	CBA, CLA	FICI = 0.5	DBM	Fadli et al., 2012
*T. riatarum*	Gentamycin	MDR *K. P. aeruginosa*	CBA, CLA	FICI = 0.18	DBM	Fadli et al., 2012
*T. riatarum*	Gentamycin	MDR *S. aureus*	CBA, CLA	FICI = 0.5	DBM	Fadli et al., 2012
*T. riatarum*	Pristinamycin	MDR *E. cloacae*	CBA, CLA	FICI = 0.5	DBM	Fadli et al., 2012
*T. riatarum*	Pristinamycin	MDR *S. aureus*	CBA, CLA	FICI = 0.5	DBM	Fadli et al., 2012
*Zataria multiflora*	Vancomycin	*S. aureus*	CBA	FICI = 0.32	–	Mahboubi and Bidgoli, 2010
Carvacrol	Ampicillin	*S. aureus*	CBA	FICI < 0.3	–	Palaniappan and Holley, 2010
Carvacrol	Bacitracin	*S. aureus*	CBA	FICI < 0.3	–	Palaniappan and Holley, 2010
Carvacrol	Nitrofurantoin	*K. oxytoca*	CBA	FICI ≤ 0.5	–	Zhang et al., 2011
Carvacrol	Ciprofloxacin	*S. aureus*	MDM, CLA	8-fold reduction in MIC	DBM	Fadli et al., 2012
Carvacrol	Ciprofloxacin	*K. pneumoniae*	MDM, CLA	4-fold reduction in MIC	DBM	Fadli et al., 2012
Carvacrol	Ciprofloxacin	*E. cloacae*	MDM, CLA	4-fold reduction in MIC	DBM	Fadli et al., 2012
Carvacrol	Tetracycline	*S. aureus* I-58	MDM	2-fold reduction in MIC	IEPA/E	Cirino et al., 2014
Eugenol	Vancomycin, β-lactams	*E. aerogenes, P. aeruginosa*	MDM, CBA	–	DBM	Hemaiswarya and Doble, 2009
Geraniol	Chloramphenicol	*E. aerogenes* EA27	MDM	256-fold reduction in MIC	IEPA/E	Lorenzi et al., 2009
*Thymol*	Tetracycline	*S. aureus* I-58	MDM	2-fold reduction in MIC	IEPA/E	Cirino et al., 2014

CBA, checkerboard assay; CLA, cell lysis assay; IZGC, change in inhibition zone by EO gaseous contact; OM, outer membrane, LPS, lipopolysaccharide structure; IEPA/E, inhibition of efflux pumps activity/expression; DBM, disruption of bacterial membrane.

In combination with chloramphenicol, several essential oils viz. *Artemisia herba-alba, Cymbopogon citratus, Helichrysum italicum*, and *Thymus riatarum* showed synergistic effects against *Enterococcus aerogenes* EA27 (overexpressing the AcrAB-TolC efflux system) (Lorenzi et al., 2009; Fadli et al., 2014, 2016). Moreover, available literature suggests that *H. italicum* and *Thymus maroccanus* act synergetically against *A. baumannii* AP1 (reduced OprD expression) (Lorenzi et al., 2009; Fadli et al., 2011). Geraniol is the major constituent in most of these oils which in combination with chloramphenicol showed synergistic effects *E. aerogenes* EA27 with 256-fold reduction in MIC (Lorenzi et al., 2009). Authors also concluded that the mechanism of action is due to inhibition of efflux pumps activity/expression (AcrAB-TolC efflux system) (Table 3). Similarly, EOs- *Thymus broussonetii* and *Thymus riatarum*, showed synergistic effects when combined with ciprofloxacin, against MDR *P. aeruginosa*, MDR *S. aureus* and MDR *K. penumoniae* Fractional inhibitory Concentration Index (FICI < 0.5) (Fadli et al., 2012). The major constituents among both essential oils are carvacrol which was also observed to be in synergism with ciprofloxacin showing 8-fold reduction in MIC. The mechanism of action is due to the disruption of the bacterial membrane (Fadli et al., 2012). Moreover, carvacrol has also been observed to be synergistic with other antibiotics, such as nitrofurantoin against *Klebsiella oxytoca* (Zhang et al., 2011), and tetracycline against MDR *S. aureus* (Cirino et al., 2014; Table 3). Gentamicin is a commonly used antibacterial agent for synergy studies in several MDR bacteria, such as *A. baumannii, S. aureus, E. clocae, P. aeruginosa*, and *K. penumoniae*. Gentamicin in combination with EOs such as *Pelargonium graveolens, T. broussonetii*, and *T. riatarum* showed synergism by action on disruption of bacterial membrane (FICI < 0.5) (Fadli et al., 2012). Fadli et al. (2012), also studied the same essential oils in combination with the antibiotic Pristinamycin against *S. aureus, E. clocae, P. aeruginosa*, and *K. penumoniae* and recorded synergistic effects (FICI = 0.5). Other major components include thymol and eugenol, whose synergistic effects were tested in combination with tetracycline (Cirino et al., 2014) and vancomycin (Hemaiswarya and Doble, 2009), respectively.

Most studies focused on bacteria *S. aureus* and *P. aeruginosa* while few studies involved *A. baumannii, Enterobacter*, and *Klebsiella.* Most essential oils are complex in nature, but few studies also followed up on the major components, e.g., carvacrol, eugenol, geraniol, and thymol. Owing to the complexity, it is important to know the mechanism of action that is responsible for synergistic effects. Table 3 describes the mechanism of actions in which mostly the disruption of bacterial membrane and inhibition of efflux pump activity/expression was observed to be the most common mode. Few studies also provide insights into the changes occuring in lipopolysaccharide structure and alteration of the outer membrane. Due to a complex mixture of components, most EOs exhibit antibacterial activities against both Gram-positive and Gram-negative pathogens. Moreover, the target bacteria and mode of action are different, as a result, the chances of resistance probability are less (Rai et al., 2017; Subramani et al., 2017). In most cases, the mode of mechanism acts as a membrane disruption, and as a result, leakage of cell content as well as coagulation of the cytoplasm. Moreover, other mechanisms involve their ability to inhibit the bacterial efflux pumps, metabolic pathways, antibiofilm, and anti-quorum sensing activity (Table 4). Moreover, due to the multiple bioactivities, such as antioxidant, anti-inflammatory, and wound healing properties, additional benefits may be observed during the treatment of ESKAPE pathogens (Subramani et al., 2017).

## *In vivo* analysis of antimicrobial properties of essential oils against ESKAPE pathogens

Biofilm-forming bacteria are commonly involved in infection of skin wounds impairing the reparative process due to prolongation of inflammatory phase (Macedo et al., 2021). Thus, the microbial infection can lead to chronic wound development (Rahim et al., 2016). In this section we discuss the application of EOs and EOCs in the treatment of wound infections provoked by biofilm-forming bacteria (Table 5) (*A. baumannii*, *P. aeruginosa*, and *S. aureus*).

**TABLE 5:** *In vivo* antimicrobial properties of essential oils against ESKAPE pathogens.

Bacteria	Essential oil/essential oil compound	Main compounds	Formulation	Model	References
*A. baumannii*	*Pimenta dioica* (L.) Merr. (Myrtaceae)	β-Myrcene (44.1%), 1,8-cineol (18.8%), Limonene (11.7%), Eugenol (8.6%)	EO was dissolved in sweet almond oil.	Excisional wounds in mice.	Ismail et al., 2020
	*Pimenta racemose* (Mill.) J.W.Moore (Myrtaceae)	β-Myrcene (39.6%), Eugenol (31%), Limonene (15.5%)	EO was dissolved in sweet almond oil.	Excisional wounds in mice.	Ismail et al., 2020
	Eugenol	–	Eugenol was dissolved in sweet almond oil.	Excisional wounds in mice.	Ismail et al., 2020
	*Origanum vulgare* (Lamiaceae)	α-Thujene, α-pinene, octanone, terpinene, p-cymene, carvacrol and thymol	–	Excisional wounds in Wistar rats.	Amini et al., 2019
*P. aeruginosa*	*Cinnamomum zeylanicum* Blume (Lauraceae)	Cinnamaldehyde (the main constituent), eugenol, coumarin, and O-methoxycinnamaldehyde.	Nanostructured lipid carrier gel containing the EO at 2% or 4%	Third-degree burn in male Sprague-Dawley rats.	Wen et al., 2021
	Cinnamaldehyde	–	–	Excisional wounds in female Swiss mice	Ferro et al., 2019
*S. aureus*	*Anethum graveolens* (Apiaceae)	α-Phellandrene (47.3%), p-cymene (18.5%) and carvone (14.1%)	Ointments containing the EO at 2 or 4%	Excisional wounds in BALB/c mice	
	*Cymbopogon citratus* (Poaceae)	Citral (38.66%), Myrcene (13.78%), Nerol (2.90%)	Carbopol gel with the EO 1%	Excisional wounds in Wistar rats	Oliveira et al., 2019
	*Oliveria decumbens* (Apiaceae)	Thymol (50.1%), γ -terpinene (20.7%), and p-cymene (17.6%)	Cream formulation containing *O. decumbens* and *P. graveolent*	Excisional wounds in BALB/c mice	Mahboubi et al., 2016
	*Pelargonium graveolent* (Geraniaceae)	β-Citronellol (39.3%) and geraniol (23.6%)			
	*Trachyspermum ammi* (L.) Sprague (Apiaceae).	Cymene (ρ) (36.64%), Terpinene (γ-) (35.98%), Thymol (18.14%)	Core-shell electrospun nanofibers containing the EO.	Excisional wounds in Sprague-Dawley male rats	Zare et al., 2021
*S. aureus* and *P. aeruginosa*	*T.carum carvi* L. (Apiaceae)	Carvone (56.9%), Limonene (36.1%)	EO encapsulated in nanostructured lipid carriers	Excisional wounds in BALB/c mice	Tazehjani et al., 2021
	*Cinnamomum verum* J.Presl (Lauraceae)	Cinnamic aldehyde (54.1%), α-copaene (12.3%), and styrene benzebe, ethenyle (7%)	Ointments containing the EO at 2 and 4%.	Excisional wounds in BALB/c mice	Seyed Ahmadi et al., 2019
	*Mentha* × *piperita* L. (Lamiaceae):	Menthol (39.80%), mentone (19.55%), neomenthol (8.82%)	Ointments containing the EO at 2, 4, or 8%	Excisional wounds in BALB/c mice	Modarresi et al., 2019
			EO encapsulated in nanostructured lipid carriers	Excisional wounds in BALB/c mice	Ghodrati et al., 2019
	*Mentha pulegium* L. (Lamiaceae)	Pulegone (72.18%), piperitenone (24.04%)	EO encapsulated in nanostructured lipid carriers	Excisional wounds in BALB/c mice	Khezri et al., 2019
	*Rosmarinus officinalis* L. (Lamiaceae)	1,8-Cineole and α-Pinene.	nanostructured lipid carrier gel containing the EO at 2% and 4%.	Excisional wounds in BALB/c mice	Khezri et al., 2019
	*Salvia officinalis* L. (Lamiaceae)	cis-Thujone (26.8%), camphor (16.4%), trans-thujone (14.1%) and 1,8-cineole (10.8%)	Ointments containing the EO at 2 and 4%.	Excisional wounds in BALB/c mice	Farahpour et al., 2020
	*Satureja sahendica* Bornm (Lamiaceae)	Carvacrol, thymol, γ-terpinene, p-cymene	Ointments containing the EO at 1, 2, and 4%.	Excisional wounds in BALB/c mice	Omarizadeh et al., 2021
	*Zataria multiflora* Boiss (Lamiaceae)	Thymol (52.90%), *p*-cymene (9.10), γ-terpinene (8.10%) and carvacrol (6.80%)	Ointments containing the EO.	Excisional wounds in BALB/c mice	Farahpour et al., 2020

### Acinetobacter baumannii

Some studies have examined the antimicrobial effects of EOs against *A. baumannii* using mammalian models. One study showed the evaluation of the EO extracted from *Origanum vulgare* (Lamiaceae) in *A. baumannii*-infected wounds in Wistar rats. The EO enhanced the healing and reduced the growth in tissue and wound secretions. The EO is mainly composed of alpha thujene, alpha-pinene, octanone, terpinene, p-cymene, carvacrol, and thymol (Amini et al., 2019).

Other research examined the *in vitro* and *in vivo* antimicrobial effects of EOs from *Pimenta dioica* (L.) Merr. and *Pimenta racemose* (Mill.) J.W.Moore (Myrtaceae) against *A. baumannii* (Premachandran and Murthy, 2022). The EOs showed *in vitro* antimicrobial and antibiofilm actions against a range of *A. baumannii* strains. The authors selected the leaf oils for *in vivo* assay using male mice. The excisional wounds were infected by two successive additions of 10 μl of *A. baumannii* suspensions (10^7^ and 10^8^ CFU/ml). The animals were treated with the EOs and eugenol dissolved in sweet almond oil (5.2 and 2 μg/ml). Interestingly, the administration of *Pimenta* EOs had the highest action than eugenol. The authors did not report data about the wound repair (Ismail et al., 2020).

### Pseudomonas aeruginosa

Trans-cinnamaldehyde is an EOC with reported antivirulence and antimicrobial action against *P. aeruginosa*. The *in vivo* antimicrobial effects of Trans-cinnamaldehyde were analyzed in wounds contaminated by *P. aeruginosa* (30 μl of 1.5 × 10^8^ CFU/ml). Female Swiss mice (4 months old) were treated by the EOC solution in Dimethylsulfoxide (DMSO). The daily administration of trans-cinnamaldehyde modulated the host inflammatory response and inhibited the bacterial growth. Interestingly, the authors reported that the EOC actions were mediated by Transient Receptor Potential Ankyrin 1 (TRPA1) (Ferro et al., 2019).

The barks of *Cinnamomum zeylanicum* (Lauraceae) bear an EO mainly composed of Cinnamaldehyde, eugenol, coumarin, and *O*-methoxycinnamaldehyde (Alizadeh Behbahani et al., 2020). Wen et al. (2021) developed and characterized topical nanostructured lipid carrier (NLC) gel loaded with *C. zeylanicum* bark EO using thermosensitive Poloxamer 407 as a gelling agent. The effectiveness of this formulation was evaluated in a model of third-degree burn in male Sprague-Dawley rats. Each wound received an injection of *P. aeruginosa* suspension (∼7.5 × 10^5^ CFU/ml). The daily administration inhibited the growth of *P. aeruginosa* at the wound tissue and enhanced the skin repair (Wen et al., 2021).

### Staphylococcus aureus

The EOs from *Oliveria Decumbens* (Apiaceae) and *Pelargonium Graveolens* (Geraniaceae) have been reported as effective against these bacteria (Mahboubi et al., 2011). These EOs were incorporated in a topical ointment and the antimicrobial effects were evaluated on MRSA-infected wounds in male Balb/c mice. In this model, the infection was established by the application of sutures containing MRSA strain. After 6 hour, the treatment was initiated and was provided three times a day. The treatment with the herbal formulation containing both EO reduced the bacterial load in the wound, with values similar to those observed in the positive control (mupirocin ointment). In addition, the EOs-incorporated ointment enhanced the healing process by collagen deposition. The major compounds detected in the EOs were thymol and β-citronellol for *O. decumbens* and *P. graveolent*, respectively (Mahboubi et al., 2016).

*Anethum graveolens* L. (Apiaceae) is popularly known as dill and its essential oil (DEO) is reported as an antimicrobial agent (Singh et al., 2005). The effects of ointments (90%, 5% hard paraffin and 5%) containing DEO at 2 or 4% were evaluated in excisional wounds infected by MRSA in male BALB/c mice (nine-weeks-old). The wounds were infected with 50 μl of MRSA suspension (5 × 10^7^CFU/wound) and treated daily with the ointment. The topical administration of DEO-ointments promoted the wound contraction and reduced the bacterial burden. Histological analysis revealed that these effects were associated with the improvement of re-epithelialization, angiogenesis and collagen deposition. The animals treated with DEO-ointments also showed higher expressions of Bcl-2, p53, caspase-3, VEGF, and FGF-2 in comparison to the untreated mice. The major compounds of DEO were α-phellandrene, p-cymene and carvone (Manzuoerh et al., 2019).

The EO from *Cymbopogon citratus* is another example of EO which reported *in vivo* anti-*S. aureus* activity. In this case, the EO was incorporated in carbopol at 1% and the formulation was applied in *S. aureus-*infected wounds (0.5 × 10^8^ CFU/wound) performed in male Wistar mice (3–4 months). The EO used is mainly composed of Citral (38.66%), Myrcene (13.78%), and Nerol (2.90%). The treatment with *C. citratus* EO reduced the bacterial load with the same effectiveness as vancomycin (Oliveira et al., 2019).

Zare et al. (2021) developed Core-shell electrospun nanofibers containing the EO from *Trachyspermum ammi* (L.) Sprague (Apiaceae) and evaluated them in wounds contaminated by *S. aureus.* In this case, the author used Sprague-Dawley male rats (7–8 weeks old) and the wounds were infected with 1,000 μl of *S. aureus* suspension (1–1.5 × 10^8^ CFU/ml). The major compounds detected in the EO were Cymene (ρ) (36.64%), Terpinene (γ) (35.98%), Thymol (18.14%). The formulation showed high efficacy in reducing the growth of *S. aureus* in the wounds and promoted the healing process (Zare et al., 2021).

### *Staphylococcus aureus* and *Pseudomonas aeruginosa*

*Mentha* × *piperita* L. (Lamiaceae) is source of an EO (known as PEO) with antibiofilm and antimicrobial actions against *S. aureus* and *P. aeruginosa* (Husain et al., 2015; Chraibi et al., 2021). The *in vivo* antimicrobial effects of ointments containing the PEO at 2, 4 and 8% were investigated. In this case, wounds in male BALB/c mice (12–14 weeks old) were infected by 25 × 10^7^ units of *S. aureus* and *P. aeruginosa*. The topical treatment with PEO-containing ointments reduced the bacterial load and the expression of some inflammatory mediators (TNF-α, VEGF and FGF-2), while the genes for CCL2, CXCL1, IL-1β, TGF-β1, and IL-10 were upregulated. On the other hand, the PEO-treated wounds showed faster wound contraction than control animals, due to the increase in fibroblasts migration and collagen synthesis (Modarresi et al., 2019). PEO loaded in nanostructured lipid carriers also exhibited efficacy in this model (Ghodrati et al., 2019).

Similarly, the antimicrobial effects of ointments prepared with *Cinnamon verum* essential oil (2 and 4%) were tested in wounds infected by *S. aureus* and *P. aeruginosa*. The ointments were prepared using soft yellow paraffin. The excisional wounds were contaminated with 10^7^ CFU of each bacterium. *C. verum* EO-containing ointment accelerated the tissue repair by inhibiting the inflammation and increasing the collagen deposition, keratin synthesis and the expression of key genes (IGF-1, FGF-2, and VEGF expression). The animals treated with *C. verum* EO exhibited higher antioxidant power. The major compounds presented in the used EO were Cinnamic aldehyde (54.1%), α-copaene (12.3%), and styrene benzebe, ethenyle (7%) (Seyed Ahmadi et al., 2019).

Other EOs that were efficient for the treatment of wounds with mixed infections by *S. aureus* and *P. aeruginosa* including those obtained from *Carum carvi* L. (Apiaceae) (Tazehjani et al., 2021), *Mentha pulegium* L. (Lamiaceae) (Khezri et al., 2019), *Rosmarinus officinalis* L. (Lamiaceae) (Khezri et al., 2019), *Salvia officinalis* L. (Lamiaceae) (Farahpour et al., 2020), *Satureja sahendica* Bornm (Lamiaceae) (Omarizadeh et al., 2021), *Zataria multiflora* Boiss (Lamiaceae) (Farahpour et al., 2020).

## Mechanism of resistance, safety and application of essential oils

The present review deals with the recent development of EOs/EOCs targeting biofilm amongst ESKAPE pathogens. Table 3 has elaborated the mode of action of selected EOs/EOCs but the gathered information is not sufficient and still, several efforts needed further studies on the mechanism of actions of individual EOs for a better understanding of the EO’s anti-biofilm mechanisms. In general majority of EO’s mechanisms of action to inhibit the formation of biofilm as well as to eradicate matured biofilm involves the action of EOs by inhibiting QS mechanisms, as well as interacting with the EPS matrix. Words and phrases like “all-natural” are safe, not correct that includes diffusing essential oils. Like other plant compounds safety of essential oils also depends largely on dosage. Many of the reported essential oils are reported to have allergic reactions to the skin as well as “hormone-related health complications” *https://www.cnet.com/health/are-essential-oils-actually-safe/.* Lu et al. (2018) studied the efficacy of oregano oil against several isolated bacteria with multidrug resistance as well as in *in vivo* burn infection model (*P. aeruginosa* PA01 and *S. aureus* USA 300). An interesting observation was found that MDR strains could not regain resistance even up to 20 passages when tested at sub-lethal concentrations. The effect of oregano oils on the skin of mice is prominent without any side effects (skin histologically or genotoxicity). Another experiment on mice model infected with *S. aureus* (ATCC #14775), aids in the survival of one-third treated population for thirty days compared to the control (all dead within 7 days) when treated with Origanum oil (Preuss et al., 2005). Yang et al. (2019), studied effects of *Artemisia vestita* oil on mice model infected with *Streptococcus pyogenes* and observed significant improvement on respiratory function of lungs as well as biochemical parameters of blood without any noticeable toxicity. Table 5, provides details of several *in vivo* studies infected with ESKAPE pathogens and most of these essential oils are effective without any side effects (Amini et al., 2019; Ferro et al., 2019; Oliveira et al., 2019; Tazehjani et al., 2021).

On the other hand, EOs are used for human kinds since ancient times. Many of the EOs are widely used in food and cosmetic as well as pharmaceutical industries without any toxicity. Indeed, it is necessary to study complete toxicity so as to make the best use of beneficial effects. The prime rewards of essential oil are its vast collection of aromatic plants (≈3000, of which 300) are known to be safe for humans by the U.S. FDA (Lu et al., 2018). Most of these essential oils are reported to possess antioxidant and antimicrobial activity, so as to consider green antimicrobials (economically low cost in crude form, biocompatible, less toxic without any harm to the environment and most importantly less resistance toward antibiotics). All these characteristics perfectly suit for an alternative effective solution for tackling antimicrobial resistance for ESKAPE pathogens. Moreover, certain limitation still needs to be addressed and more research needs to address including the “stability, selectivity, bio-availability, biocompatibility or any possible non-target or toxic effects on the human body or any type of allergy” (Yu et al., 2020).

## Conclusions, challenges, and future perspectives

Compared to the planktonic form of the ESKAPE bacteria, those found within biofilms are estimated to be up to 1,000 times less sensitive to antimicrobials. To make this scenario worse, antibiotic resistance characterizing these pathogens is an ever-growing global threat. In order to address these problems, several strategies are being explored all over the globe especially to eradicate the biofilms of these bacteria. Some strategies include antimicrobial peptides and peptoids, bacteriophages and bacteriophage-encoded products such as endolysins, compounds able to impair biofilm formation (inhibiting either biofilm structuration or the quorum-sensing mechanisms regulating the formation of the biofilm) and immunotherapies (both active and passive), also drug reuse/resensitization and drug repurposing. Despite the potential of these strategies, the most important challenge that must be faced is the possible development of bacterial antibiotic resistance. This problem may be addressed by combining these strategies among each other, as well as with conventional antibiotics. For instance, one of the most promising alternative approaches is the use of EOs since their components naturally possess antimicrobial and antibiofilm activity. EOs can mediate resensitization of the bacteria to currently available antimicrobials by inhibiting antibiotic resistance mechanisms; another example is connected to the capability of phages to disrupt the matrix of the biofilm, making it permeable to antibiotics. New combinations of different approaches must be investigated as well, in order to broaden the therapeutic options for eradication of ESKAPE-biofilm mediated infections.

## Author contributions

SP and VT: conceptualization and design and proofread of the final version. SP, SS, AB, EF, MK, LS, and VT: data curation. SP, LS, and VT: data analysis. SP, SB, SS, AB, EF, MK, LS, and VT: writing the manuscript. All authors contributed to the article and approved the submitted version.
